# Medical care-seeking patterns among women with menstrual syndromes-related diagnoses: a longitudinal population-based study

**DOI:** 10.1186/s40001-022-00871-0

**Published:** 2022-11-15

**Authors:** Jong-Long Guo, Tzu-Chi Lee, Fen-He Lin, Hsiao-Pei Hsu, Chiu-Mieh Huang

**Affiliations:** 1 Department of Health Promotion and Health Education, National Taiwan Normal University, 162, Sec. 1, He-Ping East Road, Taipei, 10610 Taiwan; 2 Department of Nursing, College of Nursing, National Yang Ming Chiao Tung University, 155, Sec. 2, Li-Nong Street, Taipei, 11221 Taiwan; 3 Institute of Clinical Nursing, College of Nursing, National Yang Ming Chiao Tung University, 155, Sec. 2, Li-Nong Street, Taipei, 11221 Taiwan

**Keywords:** Medical care-seeking patterns, Menstrual syndromes, Traditional Chinese Medicine, Western Medicine

## Abstract

**Background:**

Many women experience menstruation-related health issues during their child-bearing years. This study aimed to evaluate women’s tendency to seek Traditional Chinese Medicine (TCM) and/or Western Medicine (WM) when newly diagnosed with menstrual syndromes and to identify factors associated with their medical care-seeking behaviors.

**Methods:**

The data of a total of 47,097 women aged between 15 and 50 years with newly diagnosed menstrual syndromes in 2005 were extracted from the Taiwan National Health Insurance Database. The follow-up period was divided equally into 6 month segments over 5 years starting with patients’ first visit for obstetric/gynecologic care. Outcomes were outpatient visits and number of TCM or WM visits during each period. Patients’ tendency for medical care utilization was estimated using Poisson regression analysis.

**Results:**

Number of outpatient visits using TCM was 0.62 (29187/47097), and using WM was 1.67 (78697/47097) within 6 months after the first menstrual syndrome diagnosis. The tendency for TCM utilization increased as follow-up time increased after controlling for potential confounders, while WM utilization decreased as follow-up time increased. Age, economic status, infertility, value of prevention, baby delivery, and obstetric/gynecologic inpatient histories were significantly associated with patients’ medical care-seeking behaviors.

**Conclusions:**

TCM and WM medical care-seeking patterns are significantly different among women with diagnoses associated with menstrual syndromes. Related factors affecting medical care-seeking behavior include age, economic status, infertility, value of prevention, childbirth and Ob/Gyn inpatient histories.

## Background

Many studies have indicated that a large proportion of females at reproductive age experience menstruation-related health issues. For young women, the prevalence rates of different menstrual symptoms include dysmenorrhea 80.9 – 92.5% [1, 2], menstrual bleeding amount 19% [2], menstrual syndrome 95.8% [1, 3, 4], and premenstrual depression 5.6% [3], indicating that menstrual syndrome is the most disturbing health concern for women.

Menstrual syndrome combines the symptoms of dysmenorrhea, heavy menstrual bleeding, premenstrual syndrome, and premenstrual dysphoric disorders [5]. These symptoms may occur before menstruation or accompanying menstrual cramps and during the menstrual period itself, and also may include physical, psychological, and social aspects. Women who experience these symptoms have noticeably impaired function in their social activities or work-related performance [6]. The impact of the menstrual syndrome burden on women’s daily activities increases with symptom severity [7]. Consequently, the menstrual syndrome burden may lead to higher direct medical costs associated with increased physician visits and laboratory tests [8], and higher indirect costs through lower productivity at work [6]. The effects of menstrual syndrome vary broadly and are not restricted to socio-economic impacts.

Women who experience menstrual syndrome commonly seek medical care. Medical care-seeking behavior has been defined as any action undertaken by individuals who perceive themselves to have a health problem and tend to find an appropriate remedy [2, 9]. The health belief model [10–12] proposes that healthcare-seeking behavior is influenced by certain factors, including perceived susceptibility (subjective assessment of risk of developing a health problem), perceived severity (subjective assessment of the potential consequences of disease), benefits (the value or efficacy of engaging in a health-promoting behavior to decrease risk of disease), barriers (such as inconvenience, expense, danger, access and discomfort), cue to action (the stimulus needed to trigger the decision-making process to accept a recommended health action), and self-efficacy (confidence in one’s ability to effect change in outcomes). All of the above factors are affected by demographic, psychosocial and structural variables.

TCM has a long history in the treatment of menstrual syndrome. The World Health Organization (WHO) recognizes TCM as a legitimate medical system, and lists it in the 11th revision of the WHO International Classification of Diseases (ICD). This has helped the popularity of TCM to grow rapidly among people who are seeking alternative healthcare remedies rather than relying on WM alone. The combined use of TCM and WM, called pluralistic medical utilization or integrated medicine, is a common practice for women with menstrual syndrome in Taiwan. However, research on the utilization of TCM and WM by women with menstrual syndromes has seldom been reported, especially longitudinal population-based study, which is comparatively limited. The present study aimed to investigate medical care-seeking patterns among women with newly diagnosed conditions associated with menstrual syndromes and to identify determining factors associated with their medical care-seeking behaviors. We hypothesized that results of this study may not only provide guidance to clinicians for meeting women’s healthcare needs, but also may contribute to reducing inappropriate medical care use.

## Methods

### Data source

The National Health Insurance (NHI) program was initiated in Taiwan in 1995 and now covers nearly all Taiwan citizens. The Bureau of NHI has released all NHI data in electronic form to researchers under the National Health Insurance Research Database project (NHIRD). A representative cohort database comprising the data of 1 million people in 2005 was randomly selected from all beneficiaries by the National Health Research Institute (NHRI) for scientific purposes. The NHRI reported finding no significant differences in age, gender, or health care costs between the representative group and all beneficiaries under the NHI program. For this study, the complete WM and TCM NHI data sets were obtained from the NHIRD. The data sets contained all patients’ medical visit files, including dates, medical care facilities and specialties, patients’ genders, dates of birth, and the four major diagnoses coded in the International Classification of Disease, 9th Revision, Clinical Modification (ICD-9-CM) format.

### Study design

**Fig. 1:**
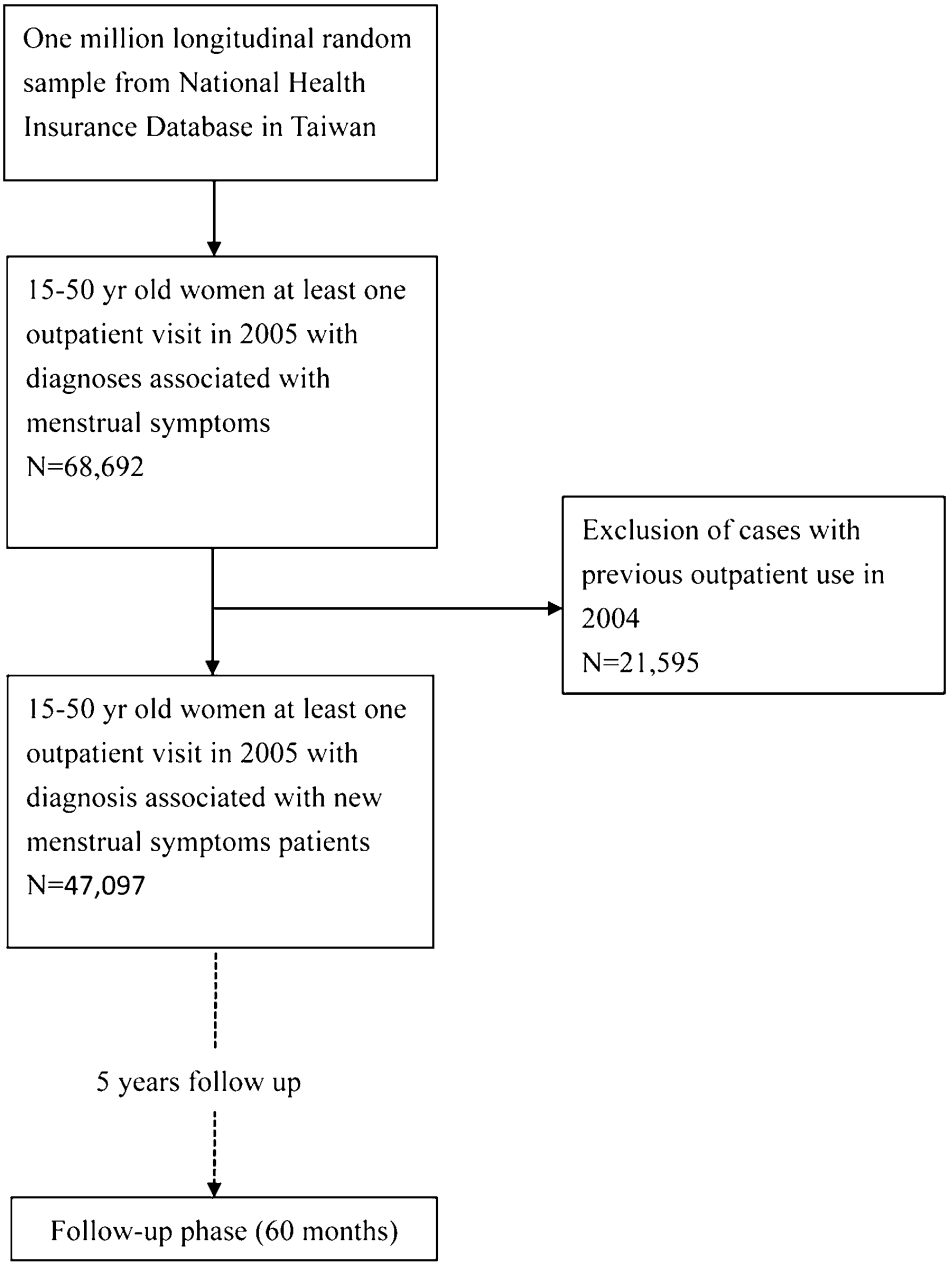
Study flowchart

In this population-based study, patient selection included all women diagnosed with menstrual syndrome in 2005 who were coded according to the ICD-9-CM. The included diagnostic categories were as follows: leiomyoma of uterus (ICD-9-CM codes 218), endometriosis (ICD-9-CM codes 617), symptoms associated with female genital organs (ICD-9-CM codes 625, except for 625.6), menstruation disorders and other abnormal bleeding from female genital tract (ICD-9-CM codes 626). All medical visit information was traced back to 2004 to confirm that these subjects were new patients. Because women may experience menstrual symptoms during their entire reproductive life cycle, women within the age range from 15 to 50 years (risk exposure) were selected. Women’s first visits to obstetricians/gynecologists/TCM in 2005 were extracted for further analysis. Medical utilization patterns for obstetric/gynecologic (Ob/Gyn) care/TCM were also followed for 5 years after their first visits. Figure 1 shows the patient selection process used in this study. Follow-up periods were divided into 10 time-periods after first visits for obstetric or gynecologic/TCM healthcare services (1–6, 7–12, 13–18, 19–24, 25–30, 31–36, 37–42, 43–48, 49–54, and 55–60 months). The outpatient visits were calculated for TCM or WM within each time period.

### Main measures

The descriptive variables included TCM or WM outpatient visits, age (15–39, 40–50), residence (urban or rural), insurance premium (proxy of economic status), infertility (Yes, No), baby delivery (Yes, No), Ob/Gyn inpatient (Yes, No) and health prevention practices (Yes, No). Monthly insurance premiums were divided into 4 levels as follows: fixed premium and dependents (requiring social welfare support, no stable salary); lower income (less than 20,000 New Taiwan Dollars [NTD] per month); middle income (20,000–39,999 NTD per month); and higher income (40,000 NTD or greater per month). Implementation of health preventive measures, such as health exam, Pap smear, or mammography, represents patient’s value of prevention.

### Outcomes

Outcomes are TCM and WM utilization visits. Patients who had received outpatient diagnoses from obstetricians/gynecologists in the Department of TCM were classified as TCM medical utilization visits, and those who received outpatient diagnoses from OB/GYN clinic departments were classified as WM medical utilization visits. The TCM and WM utilization between different follow-up time intervals were obtained by calculating mean and standard deviation of outpatient visits among women who used TCM/WM during each time interval.

### Statistical analysis

Categorical variables are presented as percentages, and continuous variables as mean (SD). Multivariate Poisson regression analysis with generalized estimating equation (GEE) was used to evaluate the tendency for use of TCM or WM obstetric/gynecologic outpatient visits. Because patients may have both TCM and WM visits for treatment within a given follow-up time interval, outcomes were reported separately as counts of outpatient visits for TCM or WM by follow-up time interval (6 months). For TCM outcome analysis, time-dependent WM outpatient visits were included in the model for control purposes, and vice versa. Counts of outpatient visits were assumed to follow Poisson distribution and the GEE was used to take the within patient correlation into account. Most descriptive variables were considered as time dependent, except for patient residence and insurance premium (measured at baseline); that is, their values were assessed by each time-period, and the dynamic effect of these time-dependent variables on response variables was estimated. The unadjusted and adjusted relative risk (RR) and 95% confidence intervals (CI) were calculated. All tests were two-sided and *P* values < 0.05 were considered statistically significant. Reported *P* values were not corrected for multiple testing. Statistical analyses and data management were performed using SAS 9.4 software (SAS Institute Inc., Cary, NC).

## Results

### Patients’ characteristics

A total of 47,097 women newly diagnosed with menstrual syndrome were included in this study. Patients’ demographic and clinical characteristics are summarized in Table 1. Patients’ mean age was 32.22 (SD, 9.15) years. About three-fourths of patients were aged 15–39 years, and 81% lived in urban areas. Nearly 53% of patients had no stable salary or belonged to the lower income group. Infertility was reported by 760 (1.61%) patients, 199 (0.42%) reported baby delivery, 977 (2.07%) patients were shown as Ob/Gyn inpatients, and 4681 (9.94%) patients implemented health preventive measures.

**Table 1:** Baseline characteristics of study patients

Variables	*n*	*%*
Age, years		
15–39	35665	75.73
40–50	11432	24.27
Age, years, mean (SD)	32.22 (9.15)	
Residence		
Rural	9029	19.17
Urban	38068	80.83
Insurance premium, NT$		
Fixed premium and dependents	6111	12.98
< 20,000	18933	40.20
20,000–39,999	14618	31.04
≥ 40,000	7435	15.79
Infertility		
Yes	760	1.61
No	46337	98.39
Baby delivery		
Yes	199	0.42
No	46898	99.58
Obstetric/gynecologic inpatient		
Yes	977	2.07
No	46120	97.93
Implementation of health preventive measures^a^		
Yes	4681	9.94
No	42416	90.06

^a^Implementation of health preventive measures, such as health exam, Pap smear, or mammography, represents patient’s value of prevention

Outpatient visits within each time interval during follow-up are summarized in Table 2. The number of TCM utilization visits was 0.69, and WM utilization visits was 1.75 within 6 months after the first menstrual syndrome diagnosis. After half a year (7–12 months), only less than 30% of the patients were still receiving follow-up, but their TCM utilization increased to 0.75, and WM utilization decreased to 1.60. The observed mean outpatient visits showed an upward trend for TCM and a downward trend for WM.

**Table 2:** Outpatient visits during the follow-up period

Time periods	No. of patients	No. of visits	Utilization^a^ [Mean (SD)]
Traditional chinese medicine			
1–6 months of follow-up	47,097	29,187	0.62 (1.80)
7–12 months of follow-up	13,544	10,091	0.75 (1.98)
13–18 months of follow-up	12,720	9585	0.75 (2.03)
18–24 months of follow-up	12,146	9937	0.82 (2.19)
25–30 months of follow-up	11,963	9647	0.81 (2.15)
31–36 months of follow-up	11,525	9798	0.85 (2.22)
37–42 months of follow-up	11,378	9967	0.88 (2.23)
43–48 months of follow-up	11,171	10,570	0.95 (2.36)
49–54 months of follow-up	10,878	10,481	0.96 (2.31)
55–60 months of follow-up	10,310	9823	0.95 (2.35)
Western medicine			
1–6 months of follow-up	47,097	78,697	1.67 (1.69)
7–12 months of follow-up	13,544	21,654	1.60 (1.67)
13–18 months of follow-up	12,720	20,817	1.64 (1.73)
18–24 months of follow-up	12,146	19,250	1.58 (1.62)
25–30 months of follow-up	11,963	19,223	1.61 (1.66)
31–36 months of follow-up	11,525	18,256	1.58 (1.64)
37–42 months of follow-up	11,378	17,810	1.57 (1.67)
43–48 months of follow-up	11,171	17,692	1.58 (1.69)
49–54 months of follow-up	10,878	17,093	1.57 (1.74)
55–60 months of follow-up	10,310	15,972	1.55 (1.69)

^a^The TCM and WM utilization among different follow-up time interval were obtained by calculating mean and standard deviation of outpatient visits among women who used TCM/WM at each time interval

### Factors affecting the tendency for TCM utilization

Multivariate Poisson regression analysis revealed that the tendency for TCM utilization increased as the follow-up time increased after controlling for potential confounders (Table 3). Younger women (aged 15–39 years), higher economic status, infertility women, and patients who were hospitalized for obstetrics and gynecology were factors that significantly increases the tendency for seeking TCM outpatient services (all *p* < 0.05). Women who had preventive health care value, and women who had baby delivery tended to reduce the use of TCM.

**Table 3:** Utilization trend of TCM gynecology clinics and the associations between TCM use and patients’ characteristics

Variables	Univariate analysis		Multivariate analysis	
	RR (95% CI)	*P* value	RR (95% CI)	*P* value
Time periods of follow-up phase, months				
1–6	[Reference]		[Reference]	
7–12	0.92^a^ (0.88–0.97)	0.002	1.17 (1.13–1.22)	** < 0.001**
13–18	0.95 (0.90–1.01)	0.076	1.18 (1.13–1.24)	** < 0.001**
18–24	1.03 (0.97–1.10)	0.284	1.25 (1.19–1.31)	** < 0.001**
25–30	1.03 (0.97–1.10)	0.267	1.24 (1.18–1.3)	** < 0.001**
31–36	1.11 (1.04–1.17)	0.001	1.29 (1.22–1.35)	** < 0.001**
37–42	1.12 (1.06–1.19)	< 0.001	1.29 (1.22–1.36)	** < 0.001**
43–48	1.22 (1.16–1.30)	< 0.001	1.40 (1.34–1.48)	** < 0.001**
49–54	1.24 (1.17–1.32)	< 0.001	1.41 (1.34–1.48)	** < 0.001**
55–60	1.23 (1.15–1.30)	< 0.001	1.39 (1.32–1.47)	** < 0.001**
Patients’ characteristics				
Age				
15–39	[Reference]		[Reference]	
40–50	0.91 (0.86–0.96)	0.001	0.93 (0.87–0.98)	**0.009**
Residence				
Rural	[Reference]		[Reference]	
Urban	1.06 (1.01–1.11)	0.020	1.02 (0.97–1.07)	0.510
Insurance premium, NT$				
Fixed premium and dependents	[Reference]		[Reference]	
< 20,000	1.10 (1.03–1.17)	0.005	1.04 (0.96–1.11)	0.336
20,000–39 999	1.24 (1.15–1.32)	< 0.001	1.13 (1.05–1.22)	**0.001**
≥ 40,000	1.37 (1.27–1.48)	< 0.001	1.24 (1.14–1.35)	** < 0.001**
Health prevention practices^b^, Yes vs. No	0.81 (0.78–0.84)	< 0.001	0.84 (0.80–0.88)	** < 0.001**
Infertility, Yes vs. No	1.28 (1.15–1.43)	< 0.001	1.49 (1.33–1.67)	** < 0.001**
Baby delivery, Yes vs. No	0.32 (0.28–0.36)	< 0.001	0.16 (0.13–0.19)	** < 0.001**
Ob/Gyn^c^ inpatient, Yes vs. No	0.62 (0.54–0.72)	< 0.001	1.16 (1.01–1.33)	**0.041**
Western medicine utilization	0.44 (0.42–0.46)	< 0.001	0.45 (0.43–0.47)	** < 0.001**

Bold values indicate statistically significant *p* values (*p* < 0.05)

^a^The utilization values in the table are the results after controlling the WM outpatient utilization during the tracking period

^b^Health prevention practices: health exam, Pap smear, or mammography

^c^TCM, Traditional Chinese medicine; Ob, Obstetric; Gyn, Gynecologic; WM: western medicine

### Factors affecting the tendency for WM utilization

Contrary to TCM utilization, the use of WM outpatient services decreased as follow-up time increased (Table 4). Women who had preventive health care value, infertility, and Ob/Gyn inpatient status were more inclined to seek WM outpatient services (all *p* < 0.05). Patients with higher economic status or who had baby delivery history tended to reduce the use of WM outpatient services (all *p* < 0.05).

**Table 4:** Utilization trend of WM gynecology outpatient clinics and the associations between WM use and patients’ characteristics

Variables	Univariate analysis		Multivariate analysis	
	RR (95% CI)	*P* value	RR (95% CI)	*P* value
Time periods of follow-up phase, months				
1–6	[Reference]		[Reference]	
7–12	0.88^a^ (0.86–0.90)	< 0.001	0.87 (0.85–0.88)	** < 0.001**
13–18	0.90 (0.89–0.92)	< 0.001	0.88 (0.86–0.90)	** < 0.001**
18–24	0.87 (0.85–0.89)	< 0.001	0.85 (0.83–0.86)	** < 0.001**
25–30	0.88 (0.86–0.90)	< 0.001	0.85 (0.83–0.86)	** < 0.001**
31–36	0.87 (0.85–0.88)	< 0.001	0.83 (0.81–0.85)	** < 0.001**
37–42	0.86 (0.84–0.88)	< 0.001	0.82 (0.81–0.84)	** < 0.001**
43–48	0.88 (0.86–0.90)	< 0.001	0.84 (0.82–0.86)	** < 0.001**
49–54	0.88 (0.86–0.90)	< 0.001	0.84 (0.82–0.86)	** < 0.001**
55–60	0.87 (0.85–0.89)	< 0.001	0.82 (0.80–0.84)	** < 0.001**
Patients’ characteristics				
Age				
15–39	[Reference]		[Reference]	
40–50	1.06 (1.05–1.08)	< 0.001	1.01 (1.00–1.03)	0.082
Residence				
Rural	[Reference]		[Reference]	
Urban	0.98 (0.96–1.00)	0.012	0.99 (0.98–1.01)	0.415
Insurance premium, NT$				
Fixed premium and dependents	[Reference]		[Reference]	
< 20 000	0.95 (0.93–0.97)	< 0.001	0.96 (0.94–0.98)	** < 0.001**
20,000–39,999	0.92 (0.90–0.94)	< 0.001	0.93 (0.91–0.95)	** < 0.001**
≥ 40,000	0.89 (0.87–0.91)	< 0.001	0.89 (0.87–0.91)	** < 0.001**
Health prevention practices^b^, Yes vs. No	1.21 (1.19–1.23)	< 0.001	1.22 (1.20–1.24)	** < 0.001**
Infertility, Yes vs. No	1.34 (1.3–1.39)	< 0.001	1.41 (1.37–1.46)	** < 0.001**
Baby delivery, Yes vs. No	0.91 (0.88–0.95)	< 0.001	0.90 (0.87–0.94)	** < 0.001**
Ob/Gyn^c^ inpatient, Yes vs. No	2.01 (1.95–2.07)	< 0.001	1.90 (1.84–1.96)	** < 0.001**
Traditional Chinese medicine utilization	0.83 (0.83–0.84)	< 0.001	0.84 (0.83–0.85)	**< 0.001**

Bold values indicate statistically significant *p* values (*p* < 0.05)

^a^The utilization values in the table are the results after controlling the TCM outpatient utilization during the tracking period.

^b^Health prevention practices: health exam, Pap smear or mammography.

^c^*WM* Western medicine, *Ob* Obstetric, *Gyn* Gynecologic, *TCM* Traditional Chinese medicine.

## Discussion

Results of the present study revealed that the patients’ tendency toward TCM utilization increased as the follow-up time increased, whereas the patients’ tendency toward WM utilization decreased as follow-up time increased. The frequency of use, treatment of disease categories, and commonly prescribed Chinese herbal formulas of TCM have been surveyed in previous studies by analyzing the NHIRD [13, 14]. The present study focused mainly on the analysis of outpatient utilization and patterns in the presented gynecological diagnoses rather than the use of TCM prescriptions. TCM utilization was only 0.62 and WM utilization was 1.67 within 6 months after the first menstrual syndrome diagnosis. Unlike some other diseases, symptoms of menstrual discomfort occur frequently and periodically. For women who experience menstrual discomfort every month, the frequency of related medical care utilization is very low. A previous study showed that most women experience some menstrual discomfort but only a few will seek medical care [15, 16]. Participants in another study indicated that professional treatment was sought only if they felt the pain was too intense and unbearable [17]. It is reasonable to conclude that patients start looking for medical services when symptoms are more severe or unbearable [13]. TCM is widely used in Taiwan and other Asian countries. As part of complementary and alternative medicine, TCM, like WM, is a scientific system supported by a complete theory [18]. For better treatment results, the concept of TCM suggests the importance of constitutional adjustments, which typically take a long time. Once patients accept the concept of TCM treatment, they continue to return to the clinic for treatment, and the number of times is often more as time progresses than at the initial starting point. TCM is used most frequently by patients who would like to decrease recurrent symptoms and relieve the uncomfortable side effects of treatment [19]. In contrast, the WM approach places more emphasis on the immediate effect that may relieve symptoms quickly. However, menstrual syndrome may recur repeatedly, and patients’ expectations may be frustrated, so the number of clinical visits tended to decrease compared with the initial starting point. This may help to explain why the trends of TCM and WM utilization show an inverted pattern. If we ask why the return rate of TCM gynecology clinics is higher than that of WM, we may speculate that many Taiwanese women believe TCM is less stimulating to the body and has a maintenance effect.

In terms of personal characteristics, women of younger ages (15–39 years) and those who had higher economic status tended to use TCM clinics. These findings are not consistent with those of previous studies. One previous study suggested that medical care-seeking is not associated with age [20]. In addition, older people are more likely to use TCM as their main form of care. However, with the recognition of Chinese medicine by WHO, younger people began to pay attention to the benefits of TCM for improving overall health status and conditioning the body, and they became more likely to actively seek ways to improve their well-being by curing the root of the problem. Therefore, more and more young people currently choose TCM as their main medical care. Another possible explanation is that older women already have more experience dealing with the menstrual syndrome. Instead of using TCM, they might use other alternative therapy or food remedies to resolve these discomforts.

According to TCM, many ways of treatment are available to deal with symptoms, including medications, acupuncture, infrared rays, and so on. Although the NHI program covers TCM and makes these services affordable to all enrollees, many items are still not reimbursable by NHI. As a result, out-of-pocket items of complementary healthcare have become obstacles for low-income patients. The present study found that higher economic status was associated with TCM use, which is consistent with the findings of previous research [21].

Longitudinal data (60 months) from the present study revealed that women with medical history of infertility or who were ever hospitalized due to Ob/Gyn disorders would likely use more clinical outpatient services of both TCM and WM. Because both TCM and WM clinical outpatient services are covered by NHI, we reasonably suggested that these women increased outpatient follow-up once they had been diagnosed with the documented medical history. Previous study also has indicated that the extension of NHI benefits coverage led to an increase in the utilization of outpatient services across all income groups among patients [22].

On the other hand, the utilization of health preventive services, such as screening tests, had different effects on the use of TCM and WM outpatient services. Women receiving screening tests, such as cervical Pap smears and mammography, were less likely to use TCM outpatient services and were more likely to use WM outpatient services. Previous studies found that patients who underwent gynecological cancer screening were more likely to experience symptom discomfort and would, therefore, visit outpatient clinics for treatment [15]. In addition, the use of preventive healthcare is a specific category of WM. Therefore, women who performed these health prevention practices were basically more likely to agree with and, therefore, seek WM healthcare services.

The present study also found that women who have baby delivery history tended to reduce the use of outpatient services. After childbirth, the uterus and cervix are enlarged, and the uterus no longer contracts excessively. When menstruation returns, the menstrual blood is discharged smoothly, and the previous discomfort of menstrual syndromes may be relieved.

### Strengths and limitations

An important strength of this study is that it used data from a comprehensive, high quality national database, which minimized discrepancies and selection bias. In addition, the use of longitudinal data allowed us to conduct long-term research and made it possible to explore the changes in long-term trends.

Nevertheless, this study has several limitations. First, this study analyzed patterns in the outpatient utilization of presented gynecological diagnoses rather than TCM prescriptions. Therefore, treatment prescriptions of Chinese medicine were not analyzed, and it was not possible to distinguish the treatment options among patients and explore the side effects of TCM treatment. Second, although the NHIRD is a large and trusted source of patient data, all analyses were retrospective, which does not rule out selection bias nor allow inferences of cause. Third, the follow-up period of the database extends until 2013, making it more difficult to reflect current reality. Improving the study may involve beginning the next stage of clinical questionnaires to collect new cases for follow-up. Fourth, patients with medical and neurological causes of psychiatric symptoms were not excluded in this study. In addition, specific patient data were unknown, such as whether patients used Chinese herbal remedies obtained directly from TCM pharmacies with or without prescriptions from licensed TCM physicians. Fifth, the study also did not include medical visits that were not covered by the NHI program. Thus, the frequency of outpatient utilization may have been underestimated. Last, patients may have used both TCM and WM or used WM first and then changed to TCM, and such patterns could not be differentiated.

Only the medical records with the defined gynecological diagnosis were included in this study, leading to the loss of patients who recovered, died, or canceled the insurance. Because there is no death or surrender insurance information in the data, it is difficult to distinguish the number of cured patients vs. death or surrender insurance patients.

## Conclusions

TCM and WM medical care-seeking patterns differ significantly among women with diagnoses associated with menstrual syndromes. An upward trend is observed for TCM and a downward trend for WM. Medical care-seeking behavior is influenced by age, economic status, infertility, value of prevention, baby delivery and Ob/Gyn inpatient histories. Findings of the present study help to increase clinicians’ understanding of patients’ medical care-seeking behavior, and to develop appropriate policies to meet their healthcare needs effectively, while also reducing inappropriate medical care use.

## Data Availability

The data that support the findings of this study are available from NHIRD but restrictions apply to the availability of these data, which were used under license for the current study, and so are not publicly available. Data are, however, available from the authors upon reasonable request and with permission of National Health Research Institutes, Taiwan. For more information, please visit https://nhird.nhri.org.tw/en/index.html.

## Abbreviations

TCM: Traditional Chinese medicine
WHO: World health organization
ICD: International classification of diseases
WM: Western medicine
NHI: The national health insurance
NHIRD: National health insurance research database project
NHRI: National health research institute
ICD-9-CM: International classification of disease, 9th revision, clinical modification
Ob/Gyn: Obstetric/gynecologic
SD: Standard deviation
NTD: New Taiwan dollars
RR: Relative risk
CI: Confidence interval
